# “Somebody’s Gonna Go Home and Put it on Facebook”: A Mixed Method Exploration of Black Youth and Adults’ Online Exposure to Violence in a Low-income Community

**DOI:** 10.1007/s40653-025-00706-0

**Published:** 2025-04-17

**Authors:** Colleen S. Walsh, Katherine M. Ross, Kiara Brown, Carine E. Leslie, Terri N. Sullivan

**Affiliations:** 1 Department of Psychology, Vanderbilt University, 230 Appleton Place, Nashville, TN USA; 2 Search Institute, 3001 Broadway Street NE #310, Minneapolis, MN 55413 USA; 3 Department of Psychology, Virginia Commonwealth University, P. O. Box 842018, Richmond, VA 23284 USA

**Keywords:** Online violence exposure, Community violence, Youth, Adults, Mixed method, Low-income communities

## Abstract

Exposure to violence has a devastating impact on youth well-being. In urban communities with high rates of violence, negative outcomes are exacerbated by co-occurring rates of poverty and lack of resources and opportunities for youth. Recent studies highlight social media as a conduit for youth violence, though understanding online *exposure* to violence for youth and adults in urban communities is relatively understudied. Research on youth and adult experiences with online violence exposure has been limited to primarily quantitative methods using predominantly White, middle class samples. This study employs a mixed methods design to examine youth and adult experiences with online exposure to violence in a low-income urban community. The quantitative sample consisted of 150 youth (*M*_age_ = 15.1, 52.7% female, 90.7% Black/African American) and 155 adults (*M*_age_ = 41.6, 84.5% female, 93.5% Black/African American; < $10,000 annually) who completed a measure of Online Exposure to Violence. The qualitative sample identified as Black/African American (100%) and consisted of 16 youth (12–17 years, 64% female) and 22 adults (26–58 years, 100% female). We employed an exploratory sequential design. Guided by the Transformation Framework, analyses identified themes specific to types and frequency of social media use, and violence seen on social media.

## Highlights

- Black youth and adults in low-income communities are exposed to community violence at high rates.
- This mixed method study characterizes experiences with high rates of online violence exposure in which they witness (e.g., shootings, deceased individuals, and physical and verbal fighting) and are victims (e.g., bullying, relational violence) to violence online.
- The Transformative Framework posed by Nesi and colleagues (2018a; 2018b) may provide a framework for understanding the ways online contexts change, amplify, and contribute to experiences with violence is important for understanding routes of violence prevention as well as the consequences of online violence exposure.

## Introduction

Chronic community violence in economically marginalized urban areas is a public health emergency with deleterious immediate (e.g., injury and death) and long-term (e.g., psychosocial outcomes) consequences (Gaylord-Harden et al., 2016; Wright et al., 2017). Urban communities experience elevated rates of community violence as a result of systemic racial discrimination (e.g., redlining, disinvestment, geographic isolation; Belgrave et al., 2021). Black youth and their families are disproportionately represented in urban communities and consequently are more likely to be exposed to community violence, compared to their White peers (Belgrave et al., 2021). Recent studies highlighted social media as a “vector for youth violence” (see Patton et al., 2014 for a review), though empirical work regarding social media and its role in impacting youth violence within urban communities is relatively unknown. In response to these gaps, this study sought to 1) characterize youth and adult community members’ experiences of online exposure to violence through qualitative and quantitative reports and 2) explore similarities and differences between qualitative and quantitative reports of exposure to violence on social media. We employ a mixed method examination of Black youth and adult community members'experiences with online exposure to violence in an urban area with high rates of poverty and (in-person) community violence. The multiple perspectives and methods used in this paper provide a deeper understanding of online violence exposure in communities that are most impacted by community-based violence and can inform the development of tailored violence prevention programs and context-specific measures of online violence exposure.

In-person violence exposure in urban, high poverty, low resource communities is repeated and ongoing, and has extreme negative impacts on youth development (Griggs et al., 2019). Between 45 and 96% of Black youth in urban communities have witnessed violence (Gaylord-Harden et al., 2011; Margolin & Gordis, 2000; Self-Brown et al., 2006), and 16% to 37% have experienced direct victimization (Farrell & Bruce, 1997; Spano & Bolland, 2013). Historically, exposure to community violence has consisted of three separate constructs including victimization, witnessing, and hearing about violence (Fowler et al., 2009), though the majority of studies traditionally focus on victimization and witnessing (see systematic review for full detail on individual studies; Miliauskas et al., 2022). More recently, the conceptualization of community violence exposure has expanded to include social media-specific violence exposure (Motley et al., 2020; Patton et al., 2014). However, research investigating youth and adult community member perspectives on community violence exposure via social media has been limited to quantitative methods using predominantly White, middle class samples (Patton et al., 2014). Research in emerging areas, such as online violence exposure in urban contexts, can benefit from multiple reporters (e.g., multiple perspectives) and mixed method approaches (e.g., rich, detailed data), and most importantly, communities that are most impacted by violence exposure.

### Relations Between Social Media and Youth Violence

Existing evaluations of social media and youth violence indicate social media and online spaces are platforms for posting messages, images, and videos detailing the perpetration and victimization of violent incidents (Patton et al., 2014). Numerous studies identified social media as a vector, or tool, for the exacerbation and canalization of youth violence (Lane, 2018; Patton et al., 2014). Patton and colleagues (2014) reviewed 56 articles exploring the ways in which social media perpetuates youth bullying/victimization, dating aggression and stalking, and gang violence. Results of the review suggest that youth violence increasingly occurs online, though this is limited by its exclusion of community violence exposure. Recent ethnographic work by Lane (2018) suggests that social media provides a platform for the continuation of community-based youth violence within a digital space, ultimately perpetuating violence both online and in the physical community. Thus, it is crucial to gain a fuller understanding of how social media may contribute to youth community violence. Lastly, only two of the reviewed studies were qualitative in nature, and socioeconomic status, age, race and ethnicity were not reported for any of the included studies (Patton et al., 2014).

Several recent qualitative and mixed method papers have explicitly examined youths’ witnessing cyberbullying as an aspect of online violence exposure (Meter et al., 2021; Mishna et al., 2022; Pepler et al., 2021). Findings from these studies discuss themes related to: (a) Canadian youths’ social roles and responsibilities as bystanders in cyberbullying (Pepler et al., 2021), (b) Canadian youth (4 th and 10 th graders), their teachers, and parents’ motivations, behaviors, and attitudes towards cyberbullying (Mishna et al., 2022), and (c) U.S. college student perceptions of cyberbullying (e.g., mixed reports of cyberbullying definition, cyberbullies using fake personas, and lack of education about cyberbullying resources (Meter et al., 2021). While important, these findings may have limited application when considering Black youth and adult community members in urban settings based on the differing sociodemographic and geographical contexts.

Recent qualitative studies reinforce the immediate concern for potential exposure to violence online by young people. Stay at home orders during the COVID- 19 pandemic and the social and political uproar in response to publicized racist events such as the murder of George Floyd led to a surge in social-media based violence (Babvey et al., 2021). Relatedly, a study of Black, currently incarcerated, young men examined the impact of seeing videos of police violence online on individual well-being, and found that seeing police violence online was significantly associated with feelings of sadness, anger and fear in this sample (Motley et al., 2020). This study underlines the prevalence and impact of certain kinds of online violence exposure. However, the findings may be bolstered with personal and contextual insight by the addition of qualitative and mixed method investigations, as well as use of multiple reporters. Given the unique attributes of economically disadvantaged neighborhoods, and the nuances of online social media use, the inclusion of qualitative data ensures that individual experiences are captured in a systematic way.

### Application of the Transformation Framework to Online Violence

Because few studies of online exposure to community violence focused on Black youth living in economically marginalized urban areas, the current study leaned on developmental theories as a guide. Due to scarcity of literature, we apply relevant developmental theory to the framing and interpretation of our results, specifically, the Transformation Framework (Nesi et al., 2018a, b) is used to organize the qualitative reports of online community violence exposure. The Transformation Framework was designed to understand the ways in which social media transforms in-person behavior (Nesi et al., 2018a). This framework states that the context and specific elements of social media transforms adolescents'experiences in five distinct ways: (1) frequency or immediacy of experiences, (2) amplification of experiences, (3) altered qualitative nature of experiences, (4) new compensatory behaviors, and (5) new and novel experiences. *Frequency or immediacy of experiences* may be increased through social media contexts by allowing various forms of content (text, photos, videos) to be posted and reposted rapidly. *Amplification of experiences* may occur through social media by adding another context for these experiences to occur, increasing the intensity and volume of in-person experiences. Social media and online contexts may *alter the qualitative nature* of an experience by changing the ways in which interactions are perceived or experienced (e.g., perceiving a comment as an attack because it is viewable by peers). Finally, social media and online contexts may facilitate new opportunities for *compensatory behaviors* (e.g., youth who do not engage in violence in-person may engage in violence online) and *novel experiences* (e.g., new subtypes of violence that may not be experienced in-person such as sharing screenshots of private messages or photos).

Previous work has applied the Transformation Framework to peer victimization to better understand the increases and changes in adolescents'interactions on social media (Nesi et al., 2018a, b). This theory may offer insight into how various features of social media transform youth and adult community members'experiences with community violence exposure. Additionally, this study is guided by the Neo-ecological Theory (Navarro & Tudge, 2023), a theoretical framework built off of Bronfenbrenner's (1977, p. 513) ecological systems theory, by considering social media and online contexts as part of an individual’s microsystem. Neo-ecological Theory directs developmental scientists to consider virtual microsystems in addition to the standard physical microsystems (e.g., school, family; Navarro & Tudge, 2023). Guided by these theoretical frameworks, this study seeks to better understand social media as a unique, proximal context that influences individual experiences. It is the authors’ belief that understanding the role of social media in exposing individuals to violence, particularly for Black families living in economically marginalized areas who are exposed to high levels of community violence, may be particularly important as they manage additional factors (e.g., poverty, access to resources and opportunities, and geographic isolation) that may exacerbate rates of online violence exposure, as well as compound the negative effects of in-person violence exposure (Lane, 2018).

## Current Study

This study used mixed methods to better understand the role that social media plays in exposing Black youth and adult community members in economically marginalized communities to violence. Reviews (Miliauskas et al., 2022; Patton et al., 2014) of online violence specify social media as a tool for youth violence perpetration and victimization, thought most studies have been limited to either qualitative or quantitative methods, primarily white, single-reporter samples and lacked focus on community context (e.g., Meter et al., 2021; Mishna et al., 2022; Pepler et al., 2021). This study builds on this literature by using a mixed method approach that foregrounds the lived experience and expertise of youth and adult community members. In line with recent maturation of developmental theory, the Transformation Framework (Nesi et al., 2018a, b) and Neo-ecological Theory (Navarro & Tudge, 2023) guide our research. The aims of this study are to 1) characterize youth and adult community members experiences of online exposure to violence through qualitative and quantitative reports and 2) explore similarities and differences between qualitative and quantitative reports of exposure to violence on social media.

## Method

### Setting and Participants

Data analyzed for this study were collected as part of a larger project that evaluated the effectiveness of a community-level youth violence prevention strategy in a mid-sized city in the Southeastern United States. Three communities (2 intervention and 1 control) were identified to take part in a community-based participatory action research strategy based on city surveillance data that indicated areas with high levels of community violence. In the greater study, youth, caregivers, and young adults participated in a community survey and qualitative interview to identify risk and protective factors related to community violence. The population in each community ranged from approximately 3,000 to 5,000 residents. The study communities were predominantly located in government subsidized housing. The majority of community members in these neighborhoods were Black or African American (96%), with 82% residing in female-headed households. Fifty-two per cent experienced unemployment, and 67% reported their income as below the Federal Poverty Level (HUD, n.d.). A full list of qualitative and quantitative sample demographics can be found in Table 1.

**Table 1:** Youth and Adult Community Member Demographics

	Adult	Youth
Variable	M(SD) or % (n)	M(SD) or % (n)
Qualitative Sample Variables (*Adult n* = *22; Youth n* = *15; N* = *37*)		
Sex assigned at birth (female)	100% (22)	53.3% (8)
Age (Mean)	41.9 (8.86)	15.1 (1.41)
Race (African American)	96% (21)	100% (15)
Number of years living in study neighborhood		
1–5 years	50% (11)	37.5% (6)
6–10 years	9.1% (2)	37.5% (6)
11–15 years	27.3% (6)	25% (4)
16–17 years	-	-
16–20 years	4.5% (1)	-
21 + years	9.1% (2)	-
Quantitative Sample Variables *(Adult n* = *155; Youth n* = *150)*		
Sex assigned at birth (female)	84.5% (131)	52.7% (79)
Age	41.62 (7.90)	15.01 (1.45)
Race (African American)	93.5% (145)	90.7% (136)
Number of years residing in public housing		
0 years	5.8% (9)	11.3% (17)
1–5 years	21.3% (33)	23.4% (35)
6–10 years	28.5% (44)	17.9% (27)
11–15 years	12.9% (20)	21.3% (32)
16–17 years	-	7.3% (11)
16–20 years	10.9% (17)	-
21 + years	14.8% (23)	-
I don’t know	1.3% (2)	12.7% (19)
Marital status		
Single, never been married	56.1% (87)	-
Married	14.2% (22)	-
In a relationship and living together	6.5% (10)	-
In a relationship, not living together	2.6% (4)	-
Legally separated	6.5% (10)	-
Divorced	10.3% (16)	-
Widowed	1.3% (2)	-
Other	1.9% (3)	-
Relationship to child participant		
Biological mother	75.5% (117)	-
Biological father	10.3% (16)	-
Stepfather	3.9% (6)	-
Stepmother	0.6% (1)	-
Adoptive mother	0.6% (1)	-
Grandmother	6.5% (10)	-
Other	2.6% (4)	-
Employment status		
Employed or self-employed full-time	27.1% (42)	-
Employed or self-employed part-time	14.2% (22)	-
Homemaker or caregiver	4.5% (7)	-
Unemployed	32.3% (50)	-
Unable to work	9.7% (15)	-
Student, not employed	0.6% (1)	-
Student, employed	0.6% (1)	-
Annual household income after taxes		
Less than $10,000	41.9% (65)	-
$10,000 to $14,999	14.8% (23)	-
$15,000 to $19,999	3.2% (5)	-
$20,000 to $24,999	5.2% (8)	-
$25,000 to $29,999	3.9% (6)	-
$30,000 to $34,999	3.9% (6)	-
$35,000 to $39,999	1.3% (2)	-
$40,000 to $44,999	0.6% (1)	-
$45,000 to $49,999	1.9% (3)	-
$50,000 or more	1.3% (2)	-
Decline to answer	18.1% (28)	-
On average, how many hours do you spend on social media (like Instagram, Facebook, Snapchat, Twitter, YouTube) every day?		
None	18.1% (28)	8% (12)
Less than 1 h per day	22.6% (35)	9.3% (14)
2 h per day	22.6% (35)	20% (30)
3 h per day	11% (17)	4.7% (7)
4 h per day	5.2% (8)	12% (18)
5 or more hours per day	12.3% (19)	37.3% (56)
Decline to answer	0% (0)	5.3% (8)
Do you have a smart phone? (yes)	77.4% (120)	75% (113)

“-” indicates information was not requested. The minimum and maximum ages for youth and adults in the qualitative sample were 12 to 17 and 28 to 78, respectively. The minimum and maximum ages for youth and adults in the quantitative sample were 12–17 and 23–65, respectively

### Data Collection Procedures

The methods for this study were approved by the University’s Institutional Review Board. It is important to note that qualitative and quantitative data collection primarily took place during the height of the COVID- 19 pandemic, as well as major racially-, socially-driven protests and activism following the murder of George Floyd, and political turmoil with a contentious national election. Further data collection details are provided subsequently.

#### Quantitative Procedures and Measures

We used combined cross-sectional survey data collected during two time frames from September 2020 to May of 2021 and July of 2021 to January 2022 in order to align with the qualitative data collection time frame. In total, cross-sectional data were collected from 150 youth and adults. The quantitative sample consisted of 150 youth (ages 12 to 17, *M*_age_ = 15.1, *SD* = 1.45, 52.7% female, 90.7% Black/African American) and 155 adults (ages 23 to 65, *M*_age_ = 41.6, *SD* = 7.9, 84.5% female, 93.5% Black/African American; < $10,000 annually). All caregivers and 95% of youth in the quantitative sample who endorsed multiple racial identities endorsed at least one of their identities as Black or African American. Adults provided informed consent, the youths’ caregivers gave permission for them to participate, and youth provided their assent. Participants were eligible to participate based on living in one of the three communities and were recruited using mailings, phone calls, and home visits.

Participants completed REDCap (Research Electronic Data Capture; Harris et al., 2009) surveys remotely using an emailed survey link, or over the phone where study staff read each question, participants’ answered questions aloud, and study staff marked the participant-selected answers. Study staff were available by phone or on Zoom to answer questions. Participants could skip any question, discontinue the survey anytime they wished, and each received $40 for their participation.

***Exposure to Violence on Social Media*** was assessed using a set of items developed to assess social media-based and online exposure to violence (i.e. sexual, verbal, and physical violence) that individuals face at the community (i.e. neighborhood, school surroundings, or town), national (i.e. within the United States), and international (i.e. outside of the United States) level. An initial pool of 12 items was developed by the scale creators (Jacobs et al., n.d.). Then, a panel of 15 raters reviewed the items and provided ratings (0 = No fit, 4 = Excellent fit) on each item, assessing the fit and psychometric shortcomings (e.g. double-barreled meaning, ambiguous, too long, too vague, wording too complex). The scale creators incorporated the feedback and specific comments. Several items were re-worded with slight grammatical changes to clarify the meaning. One item was deleted, and one item was added. The final scale included 12 items that measure verbal violence, sexual violence, threats of violence, and gun violence. Participants responded to the prompt: “The following questions ask about real-life events that you have seen on a social media site or online news source about your community (*i.e., near your home/housing complex or court, neighborhood, and/or within the City).* We are asking about real-life events, NOT fictional events in movies, TV-shows, and/or video games.” Followed by “In the past three months, how frequently/often have you seen the following events via an online news source or social media site?” Response options included 1 = *Never*, 2 = *Once*, 3 = *2–3 times*, 4 = *4–6 times*, 5 = *more than 6 times*, 6 = *not in the past 3 months, but it did happen before*, 7 = *Decline to answer*. Higher scores indicated higher levels of violent experiences. Items were analyzed using descriptive statistics. A list of items can be found in Table 2.

**Table 2:** Item-level Youth and Adult Online Exposure to Violence (Adult *n* = 155; Youth *n* = 150)

	Adult Exposure (one or more times, past 3 months)	Youth Exposure (one or more times, past 3 months)
Item	**% (n)**	**% (n)**
Someone being verbally degraded or being called hurtful names?	45.7% (71)	35.3% (53)
Hate speech or slurs used against someone?	49.6% (77)	32.6% (49)
Someone being yelled or screamed at?	56.1% (87)	42.6% (64)
Someone being sexually harassed (i.e., catcalling, unwanted sexual comments)?	21.3% (33)	18.6% (28)
Someone being sexually assaulted (i.e., any unwanted sexual contact, rape)?	16.8% (26)	12.1% (18)
Someone seriously threatened (i.e., threats of bodily harm without weapon)?	30.9% (48)	19.3% (29)
Someone threatened with a weapon?	32.9% (51)	23.4% (35)
Someone being physically hurt by someone else?	41.3% (64)	27.4% (41)
Someone being hurt by a weapon or object that is not a gun/knife (i.e., a baton, bat, or taser)?	29.7% (46)	22.7% (34)
Someone being shot?	28.4% (44)	24% (36)
Someone being stabbed?	16.1% (25)	14.8% (22)
Someone being killed by another person?	27.1% (42)	21.3% (32)

#### Qualitative Procedures & Interview Protocol

The qualitative sample was 100% Black and African American, consisted of 16 youth (12–17 years old, *M*_*age*_ = 15.1, *SD* = 1.41, 64% female) and 22 adult (26–58 years, *M*_*ag*e_ = 41.9, *SD* = 8.86, 100% female) community members. Participants were recruited through purposeful sampling procedures (Naderifar et al., 2017), with the goal of obtaining representation of youth and caregivers from both communities. Recruitment also sought representation of the various experiences and perspectives within each participant type (e.g., across age range and gender identity for youth, and across caregiving roles and caregiver age range and duration in the community for caregivers). The qualitative data was collected from the two intervention communities and not the comparison community. First, from the greater qualitative project, organization leaders and providers who served the two study communities were identified and recruited by phone or email. Those who participated in interviews were then asked for referrals that could include community members (i.e., adults and youth) (i.e., snowball sampling; Naderifar et al., 2017; see author reference for further details on sampling). Participant consent, caregiver permission, and assent were obtained prior to completing each interview. Qualitative data for this project was collected from November 2020 to May 2021, through semi-structured, one-on-one, qualitative interviews, lasting on average 1 h and 40 min. All interviews were collected virtually by Zoom or other virtual platforms and were recorded with participant consent. Similar to experiences published by qualitative experts, we found participants to be open, receptive, and often prefer conducting interviews through a virtual format (e.g. Oliffe et al., 2021), perhaps in response to safety and health concerns during the pandemic. Additionally, in response to school closures during COVID- 19, families had been provided free Wi-Fi access, eliminating many concerns related to access. Adult participants were compensated $40, and youth were compensated $25 for their participation in the interview (e.g., time).

The interview protocol used for the broader study was developed using the Community Readiness Model (CRM; Tri‐Ethnic Center for Prevention Research, 2014). The interview questions were assessed by the project staff to ensure appropriateness for both the population and context of the study. Participants’ basic demographic information was collected prior to each interview including gender, race, and age. Participants were then asked a protocol of open-ended questions. Exploratory thematic analyses identified themes specific to types and frequency of social media use, and violence seen on social media. The questions that were analyzed for the purpose of this study were (1) “*What are negative things about social media?*” and (2) “*Do you learn about violence happening in your community on social media?*”.

### Data Analyses

The current study was a secondary data analysis of a larger research project. A modified explanatory sequential design was used as the qualitative data further explain the initial quantitative findings. This design is an approach where quantitative data is collected and analyzed first, and then qualitative data is collected and analyzed second as a means of further explaining or interpreting the quantitative findings (Creswell, 2006). In this case, both sets of data were collected at a similar time point by separate study recruitment teams. The quantitative data was still assessed and analyzed first, and then used data from key informant interviews where social media and violence were discussed to learn more about the topic. The goal of this type of design is for the qualitative phase to provide a deeper understanding and context to descriptive or preliminary quantitative findings. Specific to the qualitative data, only themes pertaining to violence exposure were included in this study. Themes of information validity, privacy, and predatory adults were discussed by participants, but not included as relevant to this study, though they are included in a different study (for those results, please see Author reference).

#### Quantitative Survey Analyses

All data cleaning and analyses for the study were conducted in SPSS version 27 (IBM Corp, 2020). Data were assessed for assumptions of normality including skewness and kurtosis, which were within normal range (less than 3 *SD* above or below the mean). Descriptive statistics were used to analyze the frequency distributions, means, and standard deviations for each survey item for the overall sample and for subgroups.

#### Qualitative Interview Analyses

The coding team conducted inductive thematic analysis in NVivo version 12 (Braun & Clarke, 2012). An inductive approach allows for the prioritization of participant voice, rather than researchers developing a codebook ahead of time, or establishing codes for a preliminary set of interviews that is then applied to the remainder of interviews. The prior mentioned inductive technique is appropriate for studies where more is known about a topic. All interviews were coded at the same time and consisted of three stages of coding including open, axial, and selective, with iterative processes and constant comparison at each stage. During the open coding stage, randomly assigned pairs of coding team members independently coded participant responses to the interview question, using descriptive labels for chunks of meaningful text. This process allowed the coders to be informed only by the data available in the creation of themes and subthemes. In the axial coding phase, the pair of coders then merged their separate NVivo files together, compared coding, and came to consensus on each portion of meaningful text. In this stage, the coders worked together to establish initial themes for the interview questions by organizing codes with theme names, definitions, and parameters. The two coding team members used consensus data to develop a codebook, and then presented the codebook to the rest of the coding team, who provided feedback and suggested revisions. In the selective coding stage, the first and second authors examined theme names, definitions, parameters, and content to further deduce themes to conceptualize and develop the thematic analysis framework within a Transformation Framework (Nesi et al., 2018a).

## Results

The results are presented in several parts. First, to address Aim 1, we report the quantitative frequencies by youth and adults and then the primary qualitative themes reported by youth and adult community members. Then, to address Aim 2, we utilized an explanatory sequential design (Creswell, 2011; Creswell & Clark, 2017). Following this mixed method design, we first examined the quantitative data (item level frequencies) for online exposure to violence. Based on our initial assessment of this data, we sought further understanding of the online violence exposure phenomenon in the sample. Then, we analyzed qualitative data to bolster our understanding of the quantitative frequencies and consider the more robust narrative regarding the experience of youth and adults with online violence exposure. The explanatory sequential design bolsters the two types, and provides a more robust understanding of youth and adult community members’ online violence exposure. Specifically providing descriptive frequencies data (quantitative), as well as details and depth (qualitative) (Creswell, 2006). Finally, based on the qualitative data shared by youth and adult community members, themes are discussed initially displayed as descriptive, and are then organized within the Transformation Framework to consider how features of social media can transform the phenomena of violence exposure.

### Quantitative Frequencies

Overall, adults and youth reported a similar quantitative frequency of online violence exposure. On average, youth experienced each item of online violence exposure 2.43 times in the prior 3 months, while adults experienced each item of online violence exposure on average 2.33 times in the past 3 months. Twenty percent to 56.1% of adults reported they had experienced 10 of 12 exposure items in the past 3 months and 20% to 42.6% of youth reported they had experienced 8 of 12 exposure items in the past 3 months (see Table 2).

### Qualitative Results

Youth and adult community members were asked about negative aspects of social media and whether or not they learn about violence happening in their community online. Data were analyzed for both participant types across the two questions. Several descriptive themes emerged from the data including: (a) Quantity of exposure to negative content, (b) Sources of information or exposure to negative content, and (c) Types of Violence participants are exposed to online. To address the second aim, we identified themes specific to instances of witnessing violence and violent victimization online and positioned them in the Transformation Framework. Using this framework allows for consideration of the distinct differences between in-person and online violence exposure and how online contexts “transform” instances of violence through the five features of social media: (a) Frequency or Immediacy of Experiences, (b) Amplification of Experiences, (c) Altered Qualitative Nature of Experiences, (d) New Opportunities—Compensatory Behaviors, and (e) New Opportunities—Novel Experiences. Notably, some themes spoke to more than one of the five modalities. Through this process, we created a context-specific theoretical model of characteristics of online violence exposure.

#### Descriptive Themes of Quantity, Source, and Type of Violence Exposure Online

A total of 29 participants (80.6%) reported they learned about violence happening in their community on social media most commonly through sources including Going Live Videos (*n* = 7), Facebook (*n* = 5), Community Members Sharing Posts (*n* = 5), Community Pages (*n* = 4), News Sources (*n* = 2), Instagram (*n* = 1), and Twitter (*n* = 1). Participants discussed learning about violence because others they follow go live and post videos. They expressed the sentiment that some people would rather record violence than help the person being harmed, “*It’s like whenever something happening, you got somebody out there that’s gonna record it. Instead of trying to fix the problem, you sit there and watch the whole thing play out, recording it, and put it on Facebook. Like it's gonna make you a star or something*.” Participants also discussed six types of violent content they had been exposed to online including Interpersonal Conflict (20), Cyberbullying (14), Fights (6), Shootings (5), Guns (4), and General Violence (4). Interpersonal Conflict was discussed most frequently and encompassed relational violence such as drama, arguments, beef, conflicts, and not being understood. For example, one youth stated, “*The beef that the youth be having on the social media. Cause a lot of times they take to social media for the wrong thing. So they use it for like the beef and all that. And that is not what social media is for.”* Youth and adult community members discussed a similar Cyberbullying subtheme, that captured targeted disrespect such as talking badly about others, gossip, bullying, bringing others down, attacking others for the way they look, making fun of others, starting rumors,

“*People use it to talk about people or put other people's business out there; Uh, the second one is cyberbullying. A lot of kids get bullied on there. They do suicidal or stuff like that. And the last one is, there's not a last one to me, in my opinion, I just know that it's cyberbullying and exposure to others that makes them feel some type of way.*”

Fights and shootings were discussed within the context of live videos and posts, “*You know people go live when a shooting happen or a fight is starting*”. Additionally, one youth participant described images of guns and violence as negative, “*when people bring in guns and violence in social media, it's very negative*.”

### Transformation Framework Application to Community Violence Exposure

In this section we consider how features of social media can transform the three domains of in-person community violence exposure—Victimization, witnessing, and hearing about violence in the community, using theory and qualitative evidence (See Table 3).

**Table 3:** Transformation Framework applied to Violence Exposure, *N* = 36

	**Original Construct:**	Violence Exposure
	**Original Conceptualization:**	Violence victimization, witnessing violence, hearing about violence
**Social Media Feature**	**Transformed Experience of Violence Exposure**	
*Frequency or immediacy of experiences*	(1) High rates of report of seeing violence on social media, (2) Viewing in-person violence on social media as it’s happening in real time, the fast travel of negative and violent information, (3) Sharing in-person “beef” and “drama” on social media	
*Amplification of experiences*	(1) The broadcasting of violence through posts and live recordings, people witnessing violence in-person, (2) Choosing to broadcast it on social media rather than provide aid to the person being harmed, (3) In-person follow up about violent content that is circulating on social media	
*Altered qualitative nature of experiences*	(1) Lack of control of opportunities to view violence content on social media, (2) lack of consequences for engaging in violent behavior and doing things you would not do in person on social media	
*New opportunities- compensatory behaviors*	New and repeated opportunity to (1) engage in or be victim to violent behavior that is anonymous, lacks privacy, and is challenging to monitor, and (2) the instigation of violent behavior	
*New opportunities- novel experiences*	New and repeated opportunity to view (1) information about specific violent deaths of people from their community, (2) in-person violence and crime starting from social media issues, (3) the bodies of deceased people, (4) premeditated violence and suicide content	

Once applied, the Transformation Framework exposed the lack of distinction in concrete sub-constructs (e.g., victimization, witnessing, hearing) that have consistently been identified in quantitative conceptualizations of in-person Community Violence Exposure

#### Online Exposure to Community Violence

##### Frequency or immediacy of experiences

Social media and online contexts appear to increase the frequency or immediacy of traditional violence exposure, for example, by allowing various forms of visual, verbal, and written expressions of violence to be posted rapidly and often. The majority (77.8%) of the youth and adult community members in the qualitative sample reported having seen instances of violence online, specifically through negative comments, bullying in direct messages, and shared posts and live videos (“*Soon as somebody go live—Oh, you finding out what's going on, at whose house, how it’s happening, when it's happening.*”). Participants highlighted how quickly posts of violent content are shared between people online (“*It can reach a lot of demographics, and information travels, and it catches their attention better than talking to them*”), and for social media users who *do not* want to see or engage with these kinds of posts, they are hard to escape because of the rate at which they are posted and shared (“*lot of people share the negative stuff way faster and more than the positive stuff”*).

##### Amplification of experiences

Social media and online contexts appear to amplify the experiences of traditional violence exposure, increasing the intensity of in-person violence experiences. Participants discussed the regular broadcasting of violence through posts and live recordings specifically in regard to extreme instances of in-person violence (“*Posting what they saw or sometimes they post, if it's a dead body, they posted that up on there. Yes. They famous for that.”*). Several adults expressed concern about the normalization of these types of posts, in part because in-person community violence (e.g., shootings, murder) is so frequent in their community. Specifically, adults mentioned discomfort with people’s willingness to record and post instances of community violence rather than assist the person who has been harmed (“*I usually find out when something's wrong in my community on social media because people are always video recording or want to be the first on the scene, so they post everything and video everything. So, unfortunately a lot is seen on social media.”*). Two adults mentioned that despite their ability to avoid seeing violent posts online, often neighbors and friends will attempt to show them the post or engage them in conversation about the post in-person (“*When it be violence, I have a lot of family that's on Facebook. They will definitely ask me, “did I see this?” Or “did I see that?””*).

##### Altered qualitative nature of experiences

Social media and online contexts appear to alter the subjective experiences of violence exposure by changing the ways in which the types of violent interactions are perceived or experienced by youth and adults. Participants discussed the feelings of lack of control over how often they may view violent content on social media, as well as the lack of consequences of those who engage in posting violent content. Several participants mentioned that people are “*brave*” behind a screen, and do things they would not do in person because of the lack of consequences (“*Online you can bully people, you messing with their head. You start messing with people head in person, you gonna get smacked. You know?*”).

##### New opportunities—compensatory behaviors

Social media and online contexts appear to allow for various new experiences that might have been unlikely or entirely impossible in the absence of digital platforms. Youth and adults described new and repeated opportunities to engage in violence or be victim to violence via social media. Additionally, the ability to interact and post anonymously or with a false identity is a primary concern, and poses a challenge for monitoring, for parents and adults supporting youth. (“*Another negative is not knowing who the person is that you are talking to. These kids out here on the social media and they don’t have no idea who the person that they talking to is, just because of the picture they look like a kid, but they are actually talking to an adult*”). Participants also expressed concern that others share information they perceive as private online (“*People be, some people post stuff about their personal business that ain’t nobody’s business*”), and that there is an overall lack of privacy in online spaces (“*They whipped that camera out and that's when I’ll started deleting, unfriend you, because I don’t want to see that stuff*). Several participants also mentioned online posts discussing anticipated or premeditated in-person violence (“*People talk about violence on here, what they’re gonna do and all this*”).

##### New opportunities—novel experiences

Social media and online contexts appear to allow for new types of violence exposure or interactions that simply would be less possible offline. Within this sample, the most salient novel experience is seeing violent death or injury (“*I told you, somebody got shot on [redacted] Street, and they was standing over top of the dead body before the police even got there*.”) and learning about the violent deaths of people they know or who live in their community online (“*Like two months ago there was a girl on my page, she was down here, and I think it was on [redacted] Street, I ain’t for sure, but there was fight that broke out and the girls drove the girl drove out the house, and she on Facebook Live and you can see it*”). Participants also mentioned there are opportunities to view negative content they may be able to avoid in person, such as images of deceased individuals and suicide related content. Several participants also noted that in-person violence appears to stem from online interactions (“*Crime, sometimes crime starts from, um, Facebook or social media stuff*.”).

## Discussion

We know community violence occurs at high rates in economically marginalized, Black, urban communities (Author reference). Despite this, studies of online violence exposure have primarily excluded this population. Theory driven, multiple reporter, mixed method research provides deeper understanding of experiences with online violence exposure, in particular for a sample reporting high rates of in person violence exposure, and can inform prevention efforts.

This mixed methods study is an initial exploration of both *quantifying and describing* the online experiences of exposure to community violence for youth and adult community members living in communities that have a long-standing history and documented accounts of enduring the burden of in-person community violence exposure. The quantitative and qualitative data provide both unique and confirming evidence to deepen our understanding of an emerging, complex issue of the compounding and exacerbated experiences of violence that communities are experiencing with the permeance of digital spaces in modern society.

The findings *quantify* the experiences of online exposure to violence for youth and adults living in urban communities experiencing high rates of in-person community violence. Both quantitative and qualitative results describe the pervasiveness of online violence exposure. The most frequently reported subtype of violence was verbal violence (e.g., seeing someone being yelled/screamed at, seeing hate speeches or slurs used against someone, and seeing someone being verbally degraded or called hurtful names). Additionally, a third of participants reported seeing someone physically hurt, over a quarter reported seeing someone being hurt by a weapon (not a gun), and a quarter reported seeing someone being shot in online spaces. The rates at which participants endorsed each violent experience type is consistent with research on physical violence in that interpersonal conflict and bullying were endorsed at considerably higher rates than physical violence behavior such as fights and shootings (Farrell et al., 2014). Overall, these statistics are alarming when considering the long-term consequences of experiencing the trauma related to violence exposure (e.g., chronic physical and mental health disease; CDC, 2022) and community members experiencing this on top of high rates of in-person community violence exposure (Bishop & Chapman, 2019; Bishop et al., 2020).

It is also important to note that a higher percentage of adults reported experiencing every type of online violence experience than youth in this sample and adults, on average, reported more frequent social media use than youth. Rationale for this disparity may be related to adult and parental monitoring of their youths’ online behavior, or general surveillance of neighborhood activity. This is the first study, to our knowledge, that examines the adult experiences of violence (other than experiences of bullying) online (e.g. Mishna et al., 2022). Adults in economically marginalized communities face a host of chronic stressors on top of trying to provide resources and safety to their families in a resource deprived and often unsafe community. The added online exposure to violence has potential compounding effects on outcomes associated with intergenerational trauma (e.g. transference of mental health difficulties; Author reference). These findings highlight the importance and urgency of addressing online exposure to violence.

In addition, the findings *describe* the experiences of online exposure to violence for adults and youth. The types of violence reported are consistent with traditional in-person construct formulations of community violence exposure (e.g., including instances of hearing about violence, witnessing violence, and violence victimization). Qualitative data described instances of interpersonal conflict, cyberbullying, fights, and gun violence online. Despite the similarities of online experiences to in-person community violence exposure, this study highlights some key qualitative differences that suggest a transformative experience of *online community violence exposure* for Black youth and adults living in economically marginalized communities with high rates of community violence.

Youth and adult accounts of online exposure to violence were in line with theoretical conceptualizations of transformative experiences on digital spaces (Nesi et al., 2018a, b) that suggest social media environments may be influential in shaping behavioral processes (Patton et al., 2014). For instance, the frequency, immediacy, amplification, qualitative nature, and novel opportunities (Nesi et al., 2018a, b) for exposure to violence online makes experiences of violence nearly impossible to avoid and not constrained by location. In-person witnessing violence or violence victimization is often dependent on being in a physical location when the violent event occurs (e.g. wrong place or time), whereas online exposure can happen at any time, at any location, and to any person, regardless of their level of involvement. This is particularly true for individuals whose online social networks are of neighbors, friends, and family members who are living in the same or similar communities experiencing high rates of violence.

One difference in the qualitative nature (Nesi et al., 2018a, b) of online exposure to violence is that the distinction between witnessing violence and violence victimization (Luthar & Goldstein, 2004) is less clear. All experiences of victimization that occur via online platforms *are also witnessed*. This has implications for instances where retaliation is likely when peers or fellow community members are witness to the event. In many ways, the broadcasting, display, and sharing of community violence “ups the ante”, making violent instances less likely to fizzle or be forgotten about (Patton et al., 2014). Additionally, exposure to a given incident can happen indefinitely and repeatedly, with online posts living online indefinitely, being saved and shared (Nesi et al., 2018a, b). The impacts of this exposure cannot be understated, particularly when images and videos of traumatic events (e.g. shootings) are of people that youth and adults know as family, friends, or neighbors. The nature of these exposures warrants further context-specific explorations and more nuanced measures of exposure to violence to capture the compounding and personal nature of online violence.

Our triangulation of methods highlighted different forms of violence reported by community members; for instance, sexual assault and threatening others were not discussed in the qualitative data but were reported in the quantitative data. On the other hand, the qualitative data emphasized the effects and aftermath of violence that were not be captured in survey responses. Other studies also point to elements of online violence that this study did not capture such as the role of fake personas in cyberbullying (Meter et al., 2021) and the importance of creating more safe online spaces (Author reference). Taken together, the combination of qualitative and quantitative data points to opportunities for future measure development and the need for continued mixed methods work in communities experiencing high rates of violences to more fully understand the online context and its influence on in-person community violence exposure.

### Limitations

The findings are subject to several limitations. First, the findings in this study may not be generalizable to Black youth and adult community members in other urban, economically marginalized communities. Acknowledging this limitation is important, although the purpose of qualitative research is to emphasize the individual's experience rather than apply it to a general population. Another limitation was the sample size and the demographic makeup. Research centered on Black, low-income communities has often sampled youth and adults, as ours has, however it is also important to learn from the experiences of male caregivers and young adults, who are most frequently impacted by community violence. Additionally, the quantitative frequencies shared in this study are from a scale of online violence exposure that is still in development and has not yet been established as a valid measure. This data is limited and as such provides an important opportunity to be bolstered by the qualitative data in this study. This study is a secondary analysis of qualitative and quantitative data, and thus, the protocol and survey questions used in this study were not necessarily developed with our primary research questions in mind. Protocol questions included in this study included, *“What are negative aspects of social media?''* which did not constrain participants to discussing any particular negative aspect of social media such as violence. The second question “*Do you learn about violence happening in your community on social media*” was specific to violence but may have yielded different or more detailed answers if particular types of violence has been probed (e.g., cyberbullying, victimization, threats).

Additionally, a limitation of the current study is not knowing how people obtained violent social media content (e.g., whether or not they searched for this content or it was sent to them) which could impact their level of social media exposure. Further, we did not ask if participants may have been traveling to different states (U.S.) or international settings that may have influenced different responses to these items, thus this study is unable to account for these specific experiences. This exploratory study can be used to guide future protocol development to continue to build an empirical understanding of online exposure to violence. That said, our processes and analyses followed suit for best practices with mixed methods in secondary data analysis (Watkins, 2022).

### Implications and Future Directions

Future work may build off of the present study to further understand youth and adult community members’ exposure to violence online. It is important to develop quantitative measures of online violence exposure that capture the full extent of experiences, thus it may be helpful to include questions that address experiences in witnessing the aftermath of violence (i.e., images of deceased people, premeditated violence) as an element of exposure, as that was repeatedly discussed in the qualitative findings from this study. Additional items that may further the understanding of an individuals’ interpersonal proximity to violence or victimization by asking whether they have seen or know someone who has been violent or been a victim of violence online. Future qualitative study questions should be tailored to specifically ask about types of violence exposure, youth and adult community members beliefs/opinions about social media violence, and how in-person violence experiences are transformed by different aspects of social media (e.g., frequency, amplification). It may also be important to ask community members for recommendations for prevention of violence online and on social media (Author reference). Further insights may be provided by looking at adult and youth inter-dyadic reports (e.g., caregiver-youth pairs), or social networks of online exposure to violence. Additionally, we had several codes that were endorsed by only one or two participants which may serve as a valuable area of inquiry for further probing (e.g., plans or threats of in person violence, online discussion of premeditated violence). Finally, this study provides some evidence that exposure to localized community violence online has unique features to traditional conceptualizations of in-person exposure to violence due to the way information is spread and interacted with in online contexts. The intersection of online and offline exposure to community violence is important to understand as it may have differential or exacerbated outcomes for youth and families in communities with elevated rates of violence (Elsaesser et al., Nesi et al., 2018a, b; Patton et al., 2014).

## Conclusion

The results of this study further the understanding of the role social media plays in exposure to community violence among Black youth and adult community members residing in economically marginalized communities. The high rates of exposure to community violence that characterize this sample provide further support for exploring potential vectors (i.e., social media platforms) that may exacerbate this exposure. A mixed methods approach both highlights the high rates of community violence that characterize the current sample, and allows for the emphasis on the firsthand experience of youth and adult community members impacted by such exposure. In particular, the inclusion of multiple perspectives (e.g., youth and adult community members) provides important insight into the daily experience of living in communities impacted by community violence not typically captured by quantitative measures alone. Overall, results provide support for the triangulation of exposure to community violence on social media, suggesting the importance of considering these platforms in future studies and for intervention and prevention work.

## Data Availability

Quantitative and qualitative data sources are not available for request at this time.
